# High Refractive Index Silicone Gels for Simultaneous Total Internal Reflection Fluorescence and Traction Force Microscopy of Adherent Cells

**DOI:** 10.1371/journal.pone.0023807

**Published:** 2011-09-22

**Authors:** Edgar Gutierrez, Eugene Tkachenko, Achim Besser, Prithu Sundd, Klaus Ley, Gaudenz Danuser, Mark H. Ginsberg, Alex Groisman

**Affiliations:** 1 Department of Physics, University of California San Diego, La Jolla, California, United States of America; 2 Department of Medicine, University of California San Diego, La Jolla, California, United States of America; 3 Department of Cell Biology, Harvard Medical School, Boston, Massachusetts, United States of America; 4 Division of Inflammation Biology, La Jolla Institute for Allergy and Immunology, La Jolla, California, United States of America; Dalhousie University, Canada

## Abstract

Substrate rigidity profoundly impacts cellular behaviors such as migration, gene expression, and cell fate. Total Internal Reflection Fluorescence (TIRF) microscopy enables selective visualization of the dynamics of substrate adhesions, vesicle trafficking, and biochemical signaling at the cell-substrate interface. Here we apply high-refractive-index silicone gels to perform TIRF microscopy on substrates with a wide range of physiological elastic moduli and simultaneously measure traction forces exerted by cells on the substrate.

## Introduction

Animal tissues exhibit a broad range of stiffnesses, from <1 kPa in brain to ∼10 GPa in bone. When grown on a substrate, animal cells sense its rigidity, especially in a range corresponding to soft tissues, with elastic moduli, *E*, of 0.1–100 kPa[1], [2], [3]. Recent studies have emphasized the importance of variations of the rigidity in development[4], [5], [6], [7], tumorigenesis[3], [8], [9], and cell migration[10], [11]. Substrate rigidity sensing is mediated by cellular adhesion structures that exert traction forces on the substrate, and selective visualization of these adhesion structures is key to understanding rigidity sensing. If the elastic modulus of the substrate is sufficiently low, substrate deformations caused by the traction forces can be measured under a microscope and the cell traction forces can be reconstructed. The spatial patterns and dynamics of the traction forces provide important information on cytoskeletal tensions and the mechanisms of cell spreading, migration, and polarization [1], [12]. Because substrate deformation in a given area often results from traction forces applied at multiple adhesion points, the conversion of a map of substrate deformation into a cell traction force map is complicated, especially when the locations of the adhesion points are not known [13]. Adhesion points can be detected by employing molecular markers that are known to be recruited to cellular adhesion structures using wide-field or confocal fluorescence microscopy, but identification of adhesion points exerting traction forces can be challenging. In addition, the accurate assessment of the adhesion area, especially the detection of small adhesion points, can be difficult with these two types of microscopy because of their relatively high background level.

**Figure 1:**
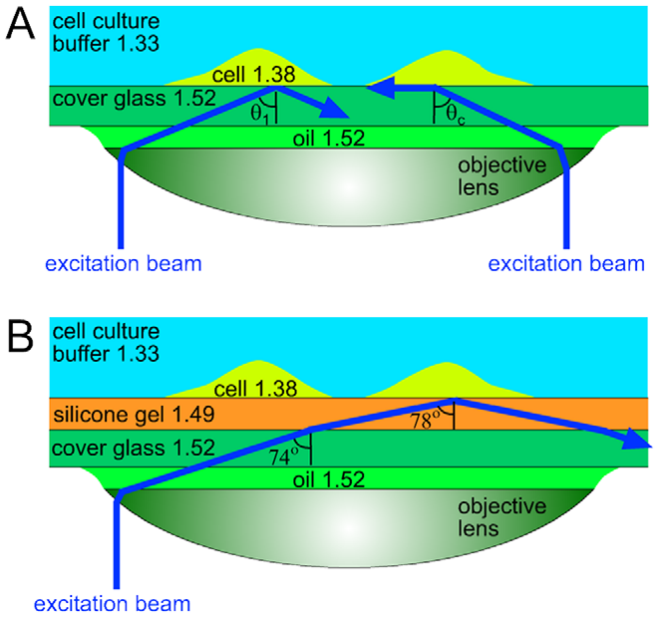
Schematic optical diagrams of through-the-lens TIRF microscopy. Excitation beams are shown in blue. Numbers indicate refractive indices of different materials found in the setup. (a) TIRF microscopy of adherent cells (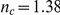) on a cover glass (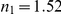). Excitation beam on the right comes at the critical angle, 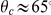. Excitation beam on the left has an angle of incidence, 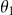, greater than 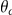. (b) TIRF microscopy of adherent cells on a thin layer of silicone gel (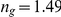) on a cover glass. Excitation beam comes at an angle 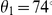, which is the maximal value achievable with an NA = 1.46 TIRF objective, is refracted at the glass-gel boundary to an angle of 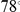, and is totally reflected at the gel-cell boundary, producing an evanescent wave with a penetration depth, 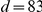
nm for 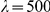
nm.

The level of fluorescence background is substantially lower in total internal reflection fluorescence (TIRF) microscopy [14], which selectively visualizes fluorescent molecules in a 100–200 nm thick layer above the substrate and is the method of choice to image the cell-to-substrate adhesion structures[15] and to study molecular trafficking events at the plasma membrane[16]. In TIRF microscopy with a popular through-the-lens illumination technique [14], the fluorescence excitation beam is directed through a marginal area of a high numerical aperture (high-NA) oil immersion objective lens. As a result, the excitation beam enters the boundary between the microscope cover glass and the medium behind it at a large angle of incidence, 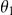 (as measured from the optical axis; Fig. 1A). If the refractive index of the medium, 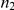, is lower than that of the cover glass, 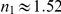, and 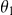 is greater than a critical angle, 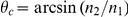, total internal reflection occurs at the boundary between the glass and the medium (Fig. 1A). The incident excitation beam is completely reflected off the boundary and only penetrates behind it as an evanescent wave that decays with the distance from the boundary, *z*, as 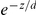, where the characteristic depth, *d*, is given by 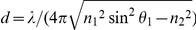, with *λ* being the wavelength of the illumination beam in vacuum [14]. (We note that 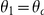 corresponds to 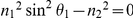 and 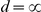.)

For a given microscope objective, there is a one-to-one correspondence between the distance of a ray from the optical axis in the plane of the objective back aperture and the resulting angle of incidence, 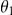 (Fig. 1). The maximal 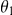 available with an objective lens is defined by the NA of the lens, 
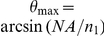
. Live cells have refractive indices of up to 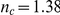 [14], [17], and for total internal reflection at the boundary between the cover glass and a cell plated on it, as required for TIRF microscopy of the cell, the condition 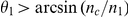 must be met (Fig. 1A). Regular oil-immersion objectives have NA up to 1.40, making them not well suited for cell TIRF microscopy, because the requirements 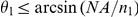 and 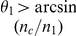 limit the range of 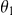 to <2° and to satisfy these requirements for all its rays, the excitation beam needs to be very narrow and collimated. In addition, 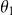 barely above 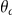 results in extended depth of the evanescent wave, *d*, (>170 nm for NA = 1.40 and *λ* = 500 nm) and the interaction of the evanescent wave with dense cellular organelles may lead to its conversion into scattered propagating light [14]. Therefore, all major manufacturers of biological microscopes introduced specialized objectives for TIRF with NA ranging from 1.45 to 1.49, greatly expanding the available range of 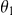 (to 7–13°), facilitating the introduction of the excitation beam, and generally improving the intensity and uniformity of illumination. Moreover, the capacities to vary 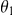 and make it substantially greater than 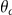 provided by specialized TIRF objectives enable adjusting *d* and making it particularly small (down to ∼70 nm with NA = 1.49, 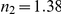, and *λ* = 500 nm) [18].

For TIRF microscopy of cells plated on a soft substrate (Fig. 1B), the refractive index of the substrate must be greater than 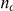. The most commonly used cell substrates that have the rigidity of soft tissue and enable traction force measurements, polyacrylamide gels [19], have a refractive index close to that of water (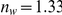), making them unsuitable for TIRF microscopy. Silicone gels made of polydimethylsyloxane (PDMS), which have a long history of use in traction force microscopy [20], [21], [22], [23], have a refractive index of 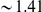. Recently, TIRF microscopy of social amoebas (*D. discoideum*) on thin layers of silicone gels with a refractive index 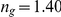 has been reported [24], [25]. In this configuration (cells on top of a gel on top of a cover glass; Fig. 1B), to reach the interface between the gel and a cell, the fluorescence excitation beam first needs to cross the interface between the cover glass and the gel, imposing a condition 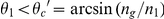 [while the condition 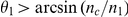 remains unchanged for TIRF at the gel-cell interface]. Rays with 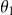 greater than the critical angle 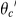 for the glass-PDMS interface will suffer a total internal reflection at this interface and not enter the gel, thus contributing to the reflection background rather than TIRF signal. Therefore, whereas the nominal NA of the objective used in the TIRF setup may be high (e.g., 1.45 as in [24], [25]), its effective NA is limited to the refractive index of the gel, 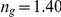 [when 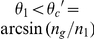 is more restrictive than 
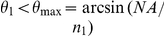
], leading to problems and limitations in the TIRF microscopy of cells similar to those before the introduction of specialized high-NA TIRF objectives.

Here we used cover glasses coated with thin layers of silicone gels with a refractive index 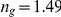 (Fig. 1B) to perform TIRF microscopy of cells on soft substrates with a specialized TIRF objective (Nikon 60x/1.49), taking full advantage of its high NA of 1.49. We formulated these high refractive index (HRI) gels with elastic moduli, *E*, of 0.4–130 kPa, covering nearly the entire physiological range, and tested their mechanical properties with a custom-built microfluidic device [26]. We plated human umbilical venous endothelial cells (HUVECs; by Lonza, Basel, Switzerland) on the gels, performed TIRF microscopy on them, and combined it with traction force microscopy by tracking fluorescent beads on surfaces of the gels.

## Materials and Methods

To prepare the gel layers, the components of the HRI gel pre-polymers, parts A and B of QGel 920 and parts A and B of QGel 903 (both by Quantum Silicones LLC, Richmond VA; refractive index of 1.49 when cured), were mixed in various proportions (Table 1) and coated onto 25 mm no. 1 round cover glasses using a home-built spin-coater rotating at 1920 rpm. Each cover glass was baked at 100°C for 2 hr to create a layer of cured gel on it with a thickness, 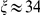 µm. After baking, the gels on the cover glasses were treated with 3-aminopropyl trimethoxysilane for 5 minutes and incubated for 10 minutes at room temperature under a suspension of 40 nm carboxylated far-red fluorescent beads (excitation/emission 690/720 nm, by Invitrogen, Carlsbad, CA) in a 100 µg/ml solution of 1-Ethyl-3-(3-dimethylaminopropyl) carbodiimide (EDC) in water to covalently link beads to the gel surface. This technique made it possible to have all beads in one plane corresponding to the surface of the gel. Therefore, the beads could be imaged under wide-field (epi-fluorescence illumination) with minimal background and their displacements reflected the deformation of the very top of the substrate. To promote cell adhesion, fibronectin (FN) was covalently linked to the gel surface by incubation in 50 µg/ml of FN with 100 µg/ml EDC in PBS, pH 7.4 for 30 min at room temperature.

**Table 1:** Relative amounts of parts A and B of QGel 920 (by mass) and parts A and B of QGel 903 (both by Quantum Silicones) used to prepare silicone gels with different elastic moduli, *E* (in kPa).

*E* (kPa)	0.4	0.7	3.7	18	30	54	130
920A	1	1	1	1	1	1	1
920B	0.95	1	1.1	2	1	1	2
903A	–	–	–	–	0.13	0.19	0.22
903B	–	–	–	–	0.13	0.19	0.22

The elastic moduli were measured with accuracies of 5–10% by applying a known shear hydrodynamic stress using a microfluidic device and measuring the resulting shear strain [26]. The gels with *E* = 0.4–18 kPa were prepared using QGel 920 only, whereas the gels with *E* = 30–130 kPa were prepared with a mixture of QGel 920 and QGel 903 components. The experiments with HUVEC were only performed on gel substrates with *E* of 0.4, 3.7, 18, and 130 kPa.

The elastic modulus (Young's modulus), *E*, of the gels was evaluated by applying a known hydrodynamic shear stress, *τ*, to the gel surface using a custom-built microfluidic device, measuring the resulting bead displacement, 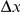, calculating the shear of the gel, 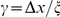, and applying the equation 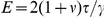, where *ν* is the Poisson ratio, as explained in detail elsewhere [26]. Because the Poisson ratio of silicone gels is nearly equal to 0.5 [27], the equation was reduced to 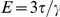. To measure the gel thickness, 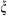, a small amount of the 40 nm far-red fluorescent beads was deposited on the cover glass surface before it was coated with the gel pre-polymer. The fluorescence microscope was first focused on beads on the glass surface and then on those on the gel surface and the difference in the readings of the nosepiece (*z*-axis) knob was recorded (with a correction for the mismatch between the refractive indices of the gel and immersion liquid), resulting in ∼1 µm accuracy. The shear, 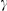, was found to be a zero-crossing linear function of *τ* for 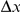 of up to at least 3 µm (greater than 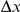
produced by HUVECs; see below) for all gels, with no sign of plastic deformations (see also [26]), thus validating the use of the equation 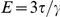, which applies to linear materials. Furthermore, measurements of 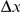
vs. 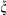 at different constant values of *τ* resulted in linear dependencies, indicating homogeneity of mechanical properties of the gel layers.

## Results and Discussion

TIRF and regular epi-fluorescence microscopy were performed on a Nikon Eclipse Ti-E microscope with a 60x/1.49 TIRF objective. To test the TIRF imaging, a suspension of fluorescent beads was pipetted onto a gel-coated cover glass. A TIRF movie (Movies S1 and S2) showed beads randomly appearing and disappearing in various locations without going out of focus, as expected for beads diffusing in and out of the thin layer above the substrate, which is illuminated under TIRF microscopy. The TIRF background of a gel-coated cover glass (with no beads) was nearly as low as the background of a blank cover glass (Fig. S1).

For live cell microscopy, HUVECs expressing lifeact-eGFP[28] were plated on the FN-coated gel substrates and incubated at 37°C in 5% CO_2_ for 1 hour. In early experiments, we observed toxic effects of freshly prepared gel substrates, which were likely due to low-molecular organic compounds present in the gels. The toxic effects disappeared, after the protocol was changed, and substrates were soaked in a buffer for ∼1 hour prior to the cell plating. A likely factor facilitating rapid elution of harmful compounds from the gels was their relatively small thickness. HUVECs were cultured on the gels for up to 3 days.

**Figure 2:**
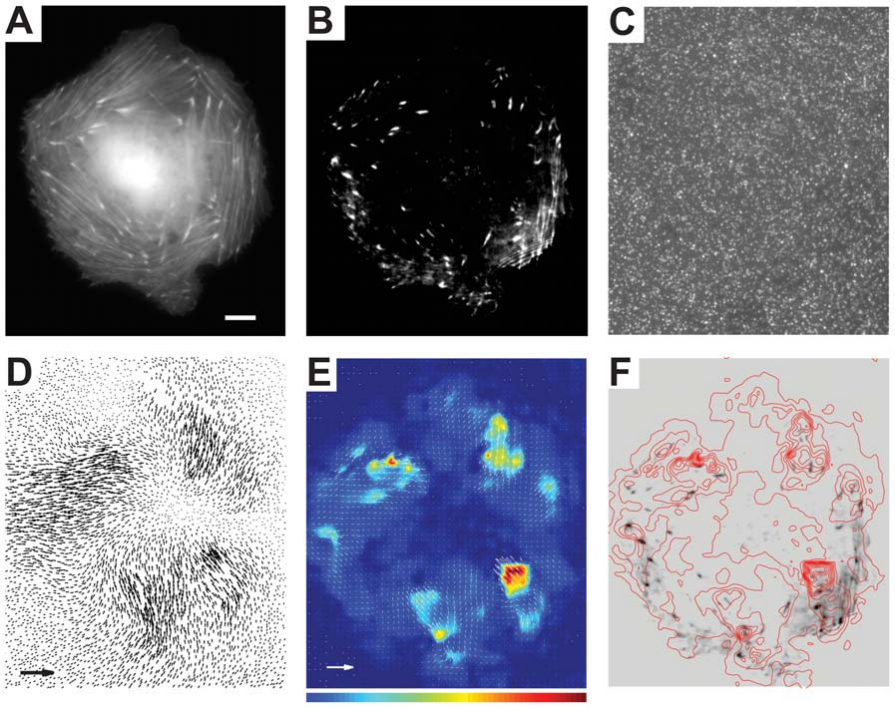
Concurrent TIRF and traction force microscopy of a HUVEC plated on a 34 µm thick layer of a silicone gel with a refractive index of 1.49 and *E* of 3.7 kPa. (a) Epi-fluorescence and (b) TIRF micrographs of fluorescently labeled F-actin in the cell. (c) Epi-fluorescence micrograph of 40nm far-red fluorescent beads covalently linked to the gel surface. (d) Vector-map of displacements of beads on the gel surface in the region shown in (a) and (b) as obtained by tracking of 40 nm far-red fluorescent beads. (e) Vector map of cell traction forces on the gel surface obtained with boundary element method [31] (white arrows) superimposed with a color-coded map of traction stress magnitudes. Blue and red ends of the spectrum correspond to cell traction stresses of 0 and 700 Pa, respectively. (f) Negative of the TIRF micrograph of the cell shown in panel B (grey and black) superimposed with a contour plot of traction stress magnitudes. Red lines connect points with identical magnitudes of cell traction stress, with 69 Pa difference in the traction stress between adjacent lines. Scale bar is 10 µm for all panels. The arrow in (d) corresponds to a displacement of 1 µm; the arrow in (e) corresponds to a traction stress of 1 kPa.

An epi-fluorescence image of a typical cell (Fig. 2A) showed a developed F-actin network, whereas in a TIRF image of the same cell, only separate fluorescent patches could be seen, corresponding to F-actin in the substrate adhesion points (Fig. 2B). Far-red fluorescent beads deposited on the substrate were imaged under epi-fluorescence illumination (Fig. 2C), first, when the cell was intact and later after it was lysed by the application of 3% Triton-X100. We note that in spite of the high surface density (6±1.7 per a 3×3 µm square), beads only protrude by 40 nm above the gel surface, occupy only ∼0.1% of the surface area, and are thus expected to have a minimal effect on cellular adhesion (less than the imprinted micro-ridge patterns proposed before [22]).

A vector map of the bead displacement due to cell traction was constructed by calculating differences between the bead positions in the first and second image using a code in Matlab (Fig. 2D)[29], [30]. The maximal bead displacement was 0.15 µm, whereas the noise level calculated as a root-mean-square of bead displacement in an area far away from the cell was 0.015 µm. A detailed reconstruction of traction forces from the substrate deformation is mathematically involved, and multiple alternative numerical protocols have been suggested [13]. Here, we used an implementation of the Boundary Element Method (BEM)[31] to convert the bead displacement map in Fig. 2D into a traction force map (Fig. 2E). The conversion was facilitated by relatively high surface density and even distribution of the tracking fluorescent beads (Fig. 2C). In addition, the placement of the beads on the surface of the gel, rather than their incorporation in the bulk, as often practiced with polyacrylamide gels [32], reduced the fluorescence background and eliminated the uncertainty regarding the vertical position of the tracer particles, resulting in more reliable conversion of displacement into stresses. (See the Supporting Information, Appendix S1, for further details on how the maps in Fig. 2D and 2E were constructed.) Superposition of a contour plot of traction stress magnitude with the TIRF image of the cell (Fig. 2F) showed many F-actin localization points in regions with high traction stresses.

**Figure 3:**
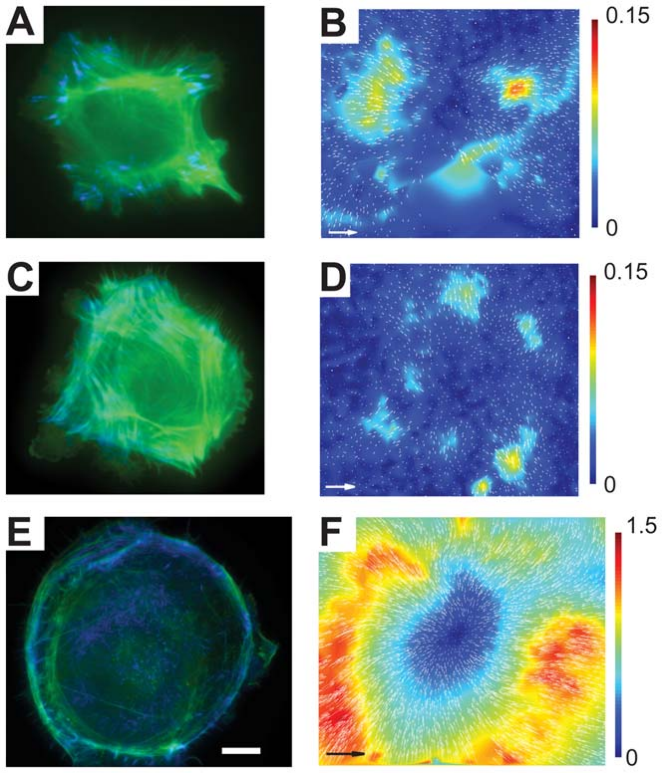
HUVECs on silicone gel substrates of various elastic moduli, *E*. (a), (c), and (e) Superimposed epi-fluorescence (false green) and TIRF (false blue) images of cells on gels with *E* = 130, 18, and 0.4 kPa, respectively. (b), (d), and (f) Arrow maps of displacements of beads on the surface of the gels by the cells in (a), (c), and (f), respectively, superimposed with color-coded maps of magnitudes of bead displacements. Bars on the right are legends for the color-coded maps with numbers indicating bead displacements in µm. Scale bar is 10 µm for all panels. Arrows in (b), (d), and (f) correspond to bead displacements of 0.3, 0.3, and 3 µm, respectively. Maximal bead displacements are ∼0.2 µm for *E* = 130 kPa, ∼0.15 µm for *E* = 18 kPa, and ∼1.8 µm for *E* = 0.4 kPa. They are somewhat higher than maximal bead displacements on the color-coded maps, because generation of the color-coded maps involved some smoothing.

The experiment was repeated with gel substrates with *E* of 0.4, 18, and 130 kPa (Fig. 3), which were prepared by mixing the components of QGel 920 and QGel 903 (that is harder than QGel 920) in different proportions (Table 1). TIRF microscopy of 4–6 cells was successfully performed on each substrate. Characteristic deformations were not visibly different for the 3.7, 18 and 130 kPa substrates, whereas characteristic traction stresses greatly increased with *E* of the substrate, in general agreement with a previous report [33]. Deformations of the gel with *E* = 0.4 kPa were substantially greater than those of the other gels (Fig. 3F vs. Figs. 3B and 3D). In addition, in contrast to cells on gels with higher *E*, cells on the gel with *E* = 0.4 kPa had substantially less actin bundles, more rounded shapes, and many F-actin localization points at the substrate (as seen with TIRF) in the central area of the cell (Fig. 3E vs. Figs. 3A, 3B and 2).

To summarize, we formulated and characterized a series of silicone gels with elastic moduli covering nearly the entire physiological range and with a refractive index of 1.49 that enables taking full advantage of the high NA oil-immersion TIRF objectives for performing TIRF microscopy of adherent mammalian cells. TIRF microscopy is expected to reduce phototoxicity, allowing improved time resolution, and because of the low background level, better resolve small adhesion points, potentially enabling the detection of single fluorescent proteins. The combination of TIRF microscopy with traction force microscopy that we demonstrated will enable studies on the contributions of various components of cell adhesion machinery to generation of substrate traction forces and on the connection between these forces and membrane trafficking events. TIRF imaging of cells grown on the silicone gels can also make it possible to relate variations in membrane trafficking events and molecular composition of adhesion points to changes in the rigidity of the substrate in a range corresponding to a variety of living tissues and pathological environments.

## Supporting Information

Figure S1 **TIRF background of a gel coated coverslip vs. blank coverslip.** (a) and (b) TIRF images of the surface of a blank coverslip and a coverslip coated with high-refractive index silicone gel, respectively, with 300×300 pixels in each image. The images were taken at identical illumination and acquisition conditions using a 100×/1.46 Olympus TIRF objective and a cooled Hamamatsu camera, with a 1 sec exposure time and a maximal gain. The images are not completely dark because of a combination of the read-out noise and dark current of the camera (amplified by the gain), the incomplete blockage of the excitation light by the fluorescence filters (combined with the reflection and scattering of light from the glass and gel surfaces), and autofluorescence of the coverslip and gel. Small dots seen on the surface of the gel are possibly due to scattering of light at small defects in the gel and residual autofluorescence of microparticles stuck to the gel surface during its preparation. (c) Histograms of the pixel values of the images in (a) and (b) are shown by red and blue dots, respectively. The mean values of the pixels were 59.5 and 64.7 for the blank and gel-coated coverslip, respectively. Therefore, the TIRF background of the gel-coated coverslip was <9% higher than that of the blank coverslip.
(TIF)

Appendix S1 **Reconstruction of the traction force map from the bead displacement map.**
(DOC)

Movie S1 **Real-time movie of a high refractive index gel substrate with a suspension of 100 nm red fluorescent beads (excitation/emission 580/605 fluorescent spheres by Invitrogen, Carlsbad, CA) above the gel taken with TIRF microscopy using a 60x/1.49 objective.** Bright objects are either steady dots, which are beads stuck to the gel surface, or less bright dots that disappear immediately after appearing (blinking), as expected for beads entering and exiting (due to their Brownian motion) an ∼100 nm deep region of evanescent illumination above the gel.
(MOV)

Movie S2 **Real-time movie of the high refractive index gel substrate with the suspension of the 100 nm red fluorescent beads above the gel (same as in Supporting movie S1) taken under wide-field fluorescence (epi-fluorescence) illumination using the 60x/1.49 TIRF objective.** In addition to steady bright dots, which are beads stuck to the gel surface, one can see two slowly moving large bright objects, which are likely aggregates of fluorescent beads, and a large number of relatively bright dots that remain visible for extended time intervals, as expected when Brownian motion of 100 nm particles is visualized under wide-field fluorescence illumination.
(MOV)
