# Supplementary figures and images for: High Refractive Index Silicone Gels for Simultaneous Total Internal Reflection Fluorescence and Traction Force Microscopy of Adherent Cells
Source: PLoS One. 2011 Sep 22;6(9):e23807. doi: 10.1371/journal.pone.0023807 (PMC3178539; doi:10.1371/journal.pone.0023807)

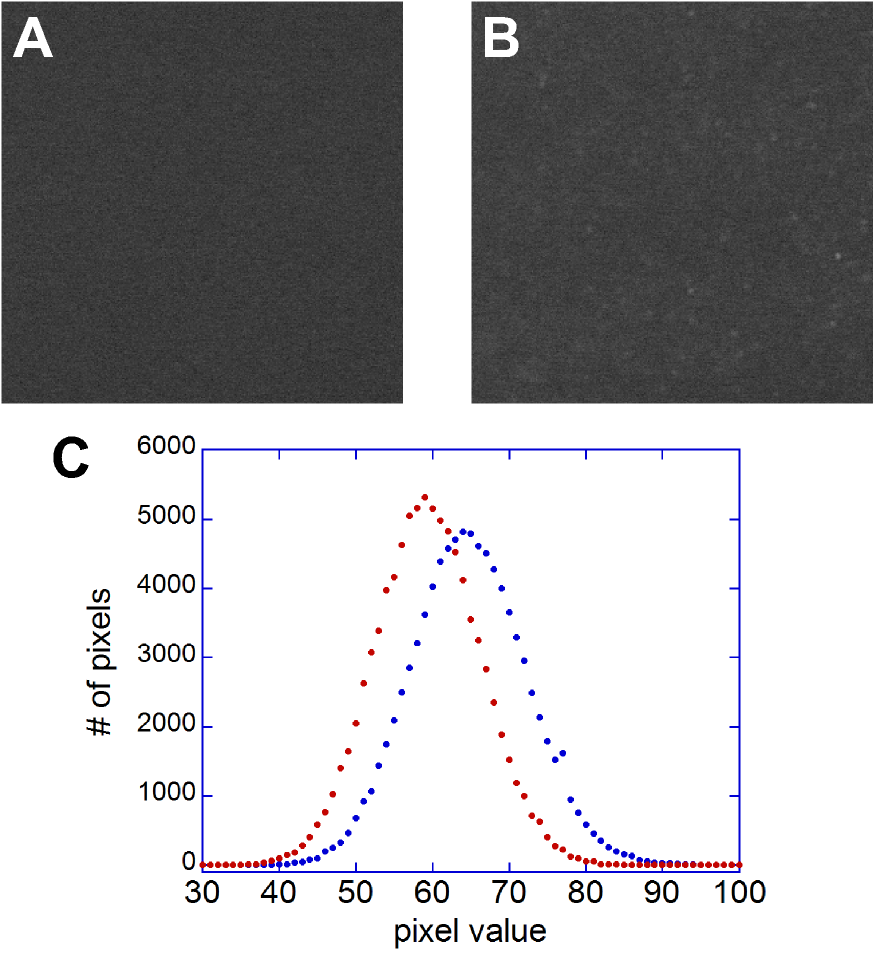

Supplement: Figure S1 — TIRF background of a gel coated coverslip vs. blank coverslip. (a) and (b) TIRF images of the surface of a blank coverslip and a coverslip coated with high-refractive index silicone gel, respectively, with 300×300 pixels in each image. The images were taken at identical illumination and acquisition conditions using a 100×/1.46 Olympus TIRF objective and a cooled Hamamatsu camera, with a 1 sec exposure time and a maximal gain. The images are not completely dark because of a combination of the read-out noise and dark current of the camera (amplified by the gain), the incomplete blockage of the excitation light by the fluorescence filters (combined with the reflection and scattering of light from the glass and gel surfaces), and autofluorescence of the coverslip and gel. Small dots seen on the surface of the gel are possibly due to scattering of light at small defects in the gel and residual autofluorescence of microparticles stuck to the gel surface during its preparation. (c) Histograms of the pixel values of the images in (a) and (b) are shown by red and blue dots, respectively. The mean values of the pixels were 59.5 and 64.7 for the blank and gel-coated coverslip, respectively. Therefore, the TIRF background of the gel-coated coverslip was <9% higher than that of the blank coverslip. (TIF) [file pone.0023807.s001.tif]
