# Supplementary material for: High Refractive Index Silicone Gels for Simultaneous Total Internal Reflection Fluorescence and Traction Force Microscopy of Adherent Cells
Source: PLoS One. 2011 Sep 22;6(9):e23807. doi: 10.1371/journal.pone.0023807 (PMC3178539; doi:10.1371/journal.pone.0023807)
Supplement: Appendix S1 — Reconstruction of the traction force map from the bead displacement map. (DOC) [file pone.0023807.s002.doc]

**Appendix S1.**

**Reconstruction of the traction force map (Figure 2E in the main text).**

Traction forces were reconstructed from the measured bead displacements using an implementation of the Boundary Element Method (BEM) originally proposed by Dembo and Wang [1]. Traction force reconstruction relies on finding the force field $\vec{f}(\vec{x}$ producing modeled substrate displacements that provides the best match to the measured bead displacement field $\vec{u}(\vec{x}$. The Green function $\mathbf{G}(\vec{x})$ is a tensor that depends on the mechanical properties of the material as well as on boundary conditions. Here, we applied the Boussinesq Green function for an isotropic, linear elastic, infinite half-space (that is applicable as long as the bead displacements are much smaller than the gel thickness). The domain of integration is fully unbiased (for example, it is not constrained by the cellular footprint) and set to the whole field of view. The reconstruction is then formulated as a least-square minimization problem:

(1)

Here, denotes the *L*_2_ norm. The first term is the deviation between the measured displacement field and the displacement field generated by the force field . Since this inversion problem is ill-posed, a second term is required for regularization [2], here formulated as zero-order Tikhonov regularization. Eq. (1) is discretized using linear triangular shape functions for the force field $\vec{f}(\vec{x}$ and then solved following the numerical scheme described by Ji *et al* [3]. The regularization parameter has been determined using the L-curve method [4]. The value used for Figure 2 is .

1. Dembo M, Wang YL (1999) Stresses at the cell-to-substrate interface during locomotion of fibroblasts. Biophysical Journal 76: 2307-2316.

2. Schwarz US, Balaban NQ, Riveline D, Bershadsky A, Geiger B, et al. (2002) Calculation of forces at focal adhesions from elastic substrate data: The effect of localized force and the need for regularization. Biophysical Journal 83: 1380-1394.

3. Ji L, Lim J, Danuser G (2008) Fluctuations of intracellular forces during cell protrusion. Nature Cell Biology 10: 1393-U1338.

4. Engl HW, Grever W (1994) Using The L-Curve For Determining Optimal Regularization Parameters. Numerische Mathematik 69: 25-31.
